# Green Synthesis of Novel Silver Nanoparticles Using *Salvia blepharophylla* and *Salvia greggii*: Antioxidant and Antidiabetic Potential and Effect on Foodborne Bacterial Pathogens

**DOI:** 10.3390/ijms25020904

**Published:** 2024-01-11

**Authors:** Addisie Geremew, John Gonzalles, Elisha Peace, Selamawit Woldesenbet, Sheena Reeves, Nigel Brooks, Laura Carson

**Affiliations:** 1 Cooperative Agricultural Research Center, Prairie View A&M University, Prairie View, TX 77446, USA; aygeremew@pvamu.edu (A.G.); jgonzales26@pvamu.edu (J.G.III); epeace1@pvamu.edu (E.P.); sewoldesenbet@pvamu.edu (S.W.); 2 Department of Chemical Engineering, College of Engineering, Prairie View A&M University, Prairie View, TX 77446, USA; smreeves@pvamu.edu (S.R.); nbrooksjr@pvamu.edu (N.B.J.)

**Keywords:** *Salvia blepharophylla*, *Salvia greggii*, antioxidant, antidiabetic, antibacterial, foodborne pathogens

## Abstract

In the face of evolving healthcare challenges, the utilization of silver nanoparticles (AgNPs) has emerged as a compelling solution due to their unique properties and versatile applications. The aim of this study was the synthesis and characterization of novel AgNPs (SB-AgNPs and SG-AgNPs, respectively) using *Salvia blepharophylla* and *Salvia greggii* leaf extracts and the evaluation of their antimicrobial, antioxidant, and antidiabetic activities. Several analytical instrumental techniques were utilized for the characterization of SB-AgNPs and SG-AgNPs, including UV–visible (UV-Vis) spectroscopy, transmission electron microscopy (TEM), dynamic light scattering (DLS), Fourier transmission infrared (FT-IR) spectroscopy, energy-dispersive X-ray analysis (EDX), and X-ray diffraction (XRD). FTIR analysis identified various functional groups in the leaf extracts and nanoparticles, suggesting the involvement of phytochemicals as reducing and stabilizing agents. High-resolution TEM images displayed predominantly spherical nanoparticles with average sizes of 52.4 nm for SB-AgNPs and 62.5 nm for SG-AgNPs. Both SB-AgNPs and SG-AgNPs demonstrated remarkable antimicrobial activity against Gram-positive bacteria *Staphylococcus aureus* and *Listeria monocytogenes* and Gram-negative bacteria *Salmonella typhimurium* and *Escherichia coli*. SB-AgNPs and SG-AgNPs also exhibited 90.2 ± 1.34% and 89.5 ± 1.5% DPPH scavenging and 86.5 ± 1.7% and 80.5 ± 1.2% α-amylase inhibition, respectively, at a concentration of 100 μg mL^−1^. Overall, AgNPs synthesized using *S. blepharophylla* and *Salvia greggii* leaf extracts may serve as potential candidates for antibacterial, antioxidant, and antidiabetic agents. Consequently, this study provides viable solutions to mitigate the current crisis of antibiotic resistance and to efficiently combat antimicrobial infections and Type 2 diabetes.

## 1. Introduction

Foodborne diseases impose a substantial and far-reaching burden on public health, economies, and societies worldwide [1,2]. Studies show that every year, one in ten individuals becomes ill due to microbial-contaminated food, resulting in a staggering 600 million illnesses, 420,000 fatalities, and the loss of 33 million healthy years of life on a global scale [2,3]. These alarming statistics underscore the urgency of addressing this global health challenge. The predominant culprits behind foodborne disease outbreaks worldwide include pathogens such as *Salmonella* spp., *Listeria* spp., and *Escherichia coli* O157 [4,5,6]. In the United States, the Centers for Disease Control and Prevention (CDC) have identified *E. coli* O157:H7 and *L. monocytogenes* as the primary foodborne pathogens responsible for numerous outbreaks [7]. Astonishingly, it is estimated that *E. coli* O157:H7 alone causes tens of thousands of infections, resulting in thousands of hospitalizations and up to one hundred deaths in the U.S. per year [8]. Clinical listeriosis, in particular, poses a grave threat, as it frequently leads to fatal outcomes, accounting for approximately 19% of all deaths attributed to major known foodborne pathogens. This represents a significant medical and public health burden [9]. However, the treatment of foodborne diseases presents an increasingly formidable challenge due to the emergence of multidrug-resistant pathogens. This trend is associated with higher mortality and morbidity rates, prolonged hospitalization periods, and significant economic losses [10,11]. Addressing this growing threat is of paramount importance to safeguard public health and minimize the socio-economic impact of foodborne illnesses.

The alarming rate of foodborne disease outbreaks has raised significant concerns about the safety of food products [3,12]. Consequently, ongoing efforts are dedicated to the development of novel and effective antimicrobial agents to combat multidrug-resistant foodborne pathogens. The convergence of bio- and nanotechnology has ushered in a transformative era in medical research, presenting unprecedented opportunities for leveraging nanomaterials across various facets of healthcare, including treatment, diagnosis, control, and modulation of biological systems [13]. In this context, nanoparticles have emerged as a promising solution for enhancing food preservation by inhibiting microbial spoilage [12]. Remarkably, due to their distinctive physical and biochemical properties, metallic nanoparticles have garnered attention in the realms of food safety, packaging, and fortification, and as potential treatments for multidrug-resistant foodborne pathogens [12,14,15]. Among the various noble metallic nanoparticles, silver nanoparticles (AgNPs) hold paramount importance in both the biomedical and food packaging industries, as potent antimicrobial, antidiabetic, and antioxidant agents [14,16,17]. AgNPs exhibit superior antimicrobial efficacy and cellular penetration when compared to their ionic counterparts [18]. Their antibacterial action is achieved through several mechanisms, including the induction of oxidative stress, disruption of membrane integrity, destabilization of essential enzymes and proteins, and inhibition of DNA replication [15,17,19]. Additionally, AgNPs have been demonstrated to disrupt cell walls and membranes in food-spoiling bacteria such as *E. coli*, *Salmonella typhimurium*, *Staphylococcus aureus*, and *L. monocytogenes*, leading to the leakage of proteins and polysaccharides [17]. These multifaceted attributes position AgNPs as a promising tool for enhancing food safety and preventing the proliferation of foodborne pathogens.

Nevertheless, the efficacy of AgNPs as versatile agents with antimicrobial, antioxidant, and antidiabetic properties depends on various factors, including their synthesis method and the physical (shape, size, morphology) and chemical properties (concentration, agglomeration, dissolution rate, functional groups attached, etc.) of the nanoparticles [17,20,21]. In particular, the use of green synthesis techniques for AgNPs offers distinct advantages over conventional methods. Green synthesis involves the use of environmentally benign solvents and non-toxic reducing agents obtained from plants, bacteria, and fungi, resulting in the fabrication of stable nanoparticles [20,22]. Recent studies have highlighted the superior antimicrobial properties of biosynthesized AgNPs using plant extracts as both reducing and capping agents when compared to chemically mediated synthesis [12,17,23]. In addition to antimicrobial activity, biosynthesized AgNPs have exhibited promise in managing diabetes by enhancing glucose uptake in cells, inhibiting α-amylase, and scavenging free radicals to prevent cellular damage [24,25,26,27]. However, due to variations in metabolite compositions and content in the plant extracts used during the reduction process, the properties and biological activities of these nanoparticles may differ [17], necessitating further exploration of potent AgNPs using a wider range of plants.

Along this vein, the utilization of medicinal plant extracts, rich in vital bioactive compounds, as reducing agents has shown promise for the synthesis of AgNPs [17,28,29]. Among medicinal plants, the sage (Salvia) genus, belonging to the Lamiaceae family, encompasses over 1000 species renowned for their culinary, cosmetic, repellent, traditional medicinal, antioxidant, and natural preservative properties [18,30,31]. In traditional medicine, Salvia species have been employed in the treatment of various ailments [21]. Furthermore, extensive in vitro experiments have revealed a broad array of pharmacological activities associated with Salvia species, including antimicrobial, antifungal, antioxidant, anti-inflammatory, anticancer, hypoglycemic, hypolipidemic, and memory-enhancing effects [21,32]. These health-promoting potentials are attributed to the diverse array of bioactive secondary metabolites abundantly present in these plants, including flavonoids, catechin, gallic acid, kaempferol, apigenin, quercetin, naringin, hypericin, luteolin, pseudohypericin, hyperforin, glycosides, rosmarinic acid, carnosic acid, carnosol, alpha-pinene, camphene, limonene, γ-terpinene, 1,8-cineol, camphor, and isobornyl acetate [21,28,30,33,34]. The potential of such diverse phytochemicals as stabilizers or capping agents during the synthesis of AgNPs has been reported in different sages, including *Salvia verticillata* [32], *S. aethiopis* [21], *S. africana-lutea* [35], *S. pratensis* [34], and *S. officinalis* [33,36,37,38,39,40]. These investigations have revealed remarkable variations in the physicochemical properties and biological applications of AgNPs. This encourages in-depth exploration of other related species, such as *S. greggii* and *S. blepharophylla*, that may hold promise for applications in both food safety and biomedical research.

The present study aimed to synthesize AgNPs using leaf extracts from the medicinal plants *S. greggii* and *S. blepharophylla* and to characterize the AgNPs and evaluate their antibacterial, antioxidant, and antidiabetic activities. This research marks the pioneering effort in the synthesis of AgNPs utilizing *S. greggii* and *S. blepharophylla* extracts, coupled with a comprehensive evaluation of their antimicrobial, antioxidant, and antidiabetic activities. The unique contribution of this study lies in its exploration of the potential applications of AgNPs, particularly those stabilized by the phytochemicals present in *S. greggii* and *S. blepharophylla* with promising prospects for utilization in the food safety and pharmaceutical industry.

## 2. Results and Discussion

### 2.1. Nanoparticle Synthesis and Characterization

**Figure 1:**
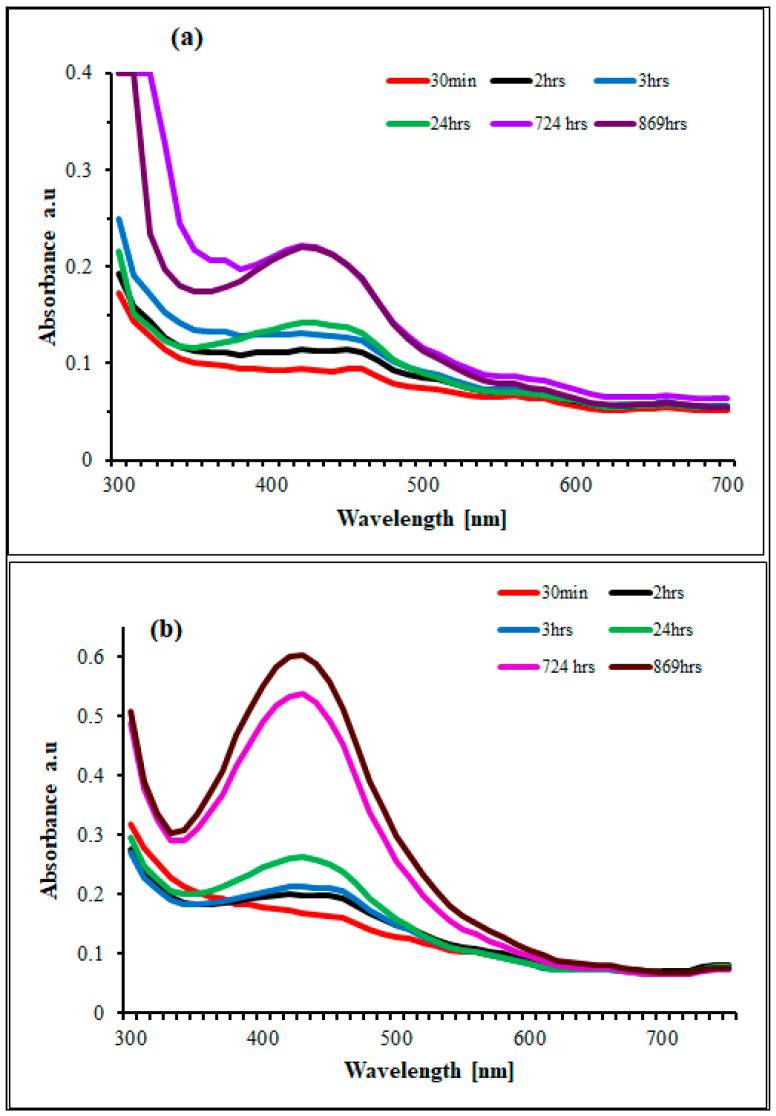
UV-Vis absorption spectrum of biosynthesized SG-AgNPs (**a**) and SB-AgNPs (**b**) at various time intervals.

The biogenic synthesis of SB-AgNPs and SG-AgNPs was confirmed through visual inspections of color change in solutions. After mixing *S. blepharophylla* and *S. greggii* leaf extracts (SBLE and SGLE, respectively) each with silver nitrate solution, a visible color change from pale yellow to dark brown resulted within 24 h. This change is the visual signal for the conversion of silver nitrate into silver nanoparticles [17,24]. In conjunction with visual assessments, UV-Vis spectroscopy analysis was used to explore the optical properties and long-term stability (from 30 min to 869 h) of SB-AgNPs and SG-AgNPs. The analysis revealed robust and broad-spectrum absorbance in the 300 to 500 nm range along with a pronounced and intense surface plasmon resonance (SPR) peak at approximately 420 nm for both SB-AgNPs and SG-AgNPs (Figure 1a,b). In agreement with our observation, *S. verticillate* and *S. officinalis* leaf extracts-mediated synthesized AgNPs demonstrated maximum absorbance in this wavelength range [32,33,34]. In contrast, Gecer [21] reported a maximum absorption in this range at 508 nm using *S. aethiopis*. The SPR might be attributed to the collective resonance of the conduction electrons in the metal [24,41]. The distinctive signature of the SPR phenomenon in AgNPs is manifested by the UV–visible absorption peak, typically occurring within the wavelength range of 300 nm to 500 nm [42]. This observation aligns with Ansar et al. [43], who demonstrated a strong absorption peak of about 420 nm in AgNPs synthesized using *Brassica oleracea* plant extracts. Concurrently, UV-Vis spectra analysis offers valuable insights into the morphological attributes of biosynthesized AgNPs, as the presence of a single SPR peak in the spectra is indicative of isotropic and spherical silver nanoparticle formations [44,45,46]. Moreover, while larger nanoparticles exhibit increased scattering leading to broader and shifted peaks toward longer wavelengths, the smaller nanoparticles primarily absorb light, resulting in peaks centered around 420 nm [34,47].

**Figure 2:**
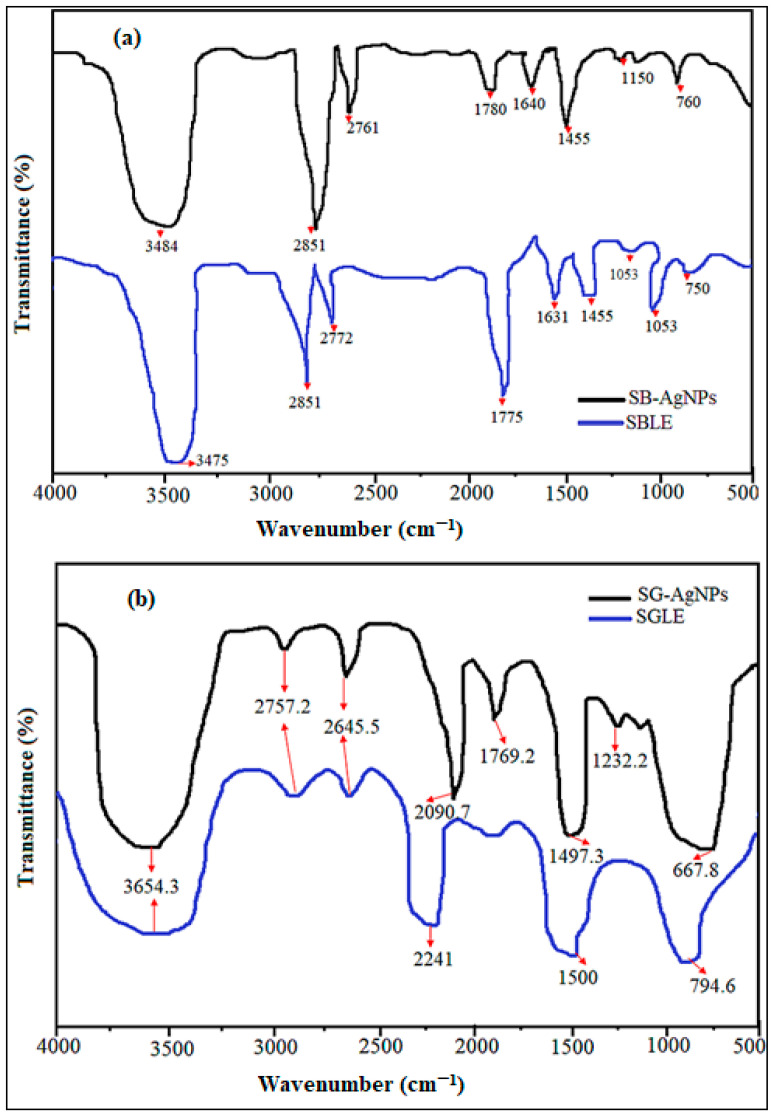
Comparison of the FTIR spectra of the *S. blepharophylla* leaf extract (SBLE) and SB-AgNPs (**a**) and *S. greggii* leaf extract (SGLE) and SG-AgNPs (**b**).

The Fourier transform infrared (FTIR) spectrum of SBLE, SGLE, SB-AgNPs, and SG-AgNPs are presented in Figure 2a,b. Peaks of SBLE and SB-AgNPs showed bands at 3475, 2851, 2772, 1775, 1631, 1455, 1053, 1052, and 750 cm^−1^ and at 3484, 2851, 2761, 1780, 1640, 1455, 1150, and 760 cm^−1^, respectively. On the other hand, the peaks of SGLE leaf extract and SG-AgNPs revealed transmittance bands at 3654, 2757, 2645, 2241, 1500, and 7945 cm^−1^ and at 3654, 2757, 2645, 2091, 1497, 1232, and 668 cm^−1^, respectively. According to Geremew et al. [17] 3000–3600 cm^−1^ peaks linked with the hydroxyl (OH) and amine (NH_2_) stretching vibrations in both plant extract and nanoparticles. The peaks between 2761 and 2918 cm^−1^ were assigned to C–H stretching of the methyl groups [48]. The vibration between 1605 and 1780 cm^−1^ of the extracts and the nanoparticles confirmed the presence of C=O stretching, corresponding to the carboxyl group [49]. The peak ranges from 1350 to 1500 cm^−1^, corresponding to the vibrations of the C–N stretch and the protein amide I band in both the extract and Ag NPs [50]. The FTIR absorption peaks of SG-AgNPs at 1232 cm^−1^ and SB-AgNPs at 1150 cm^−1^ showed N–O stretching. The peak of SB-AgNPs at 1053 cm^−1^ represents –C–O groups from the flavones and polysaccharides [51]. The peaks observed at 668, 750, 760, and 795 indicated C–H bending corresponding to aromatic compound [52] in SG-AgNPs, SBLE, SB-AgNPs, and SGLE, respectively.

Hydroxyl bond stretching in the FTIR analysis suggests the involvement of various phytochemicals such as phenols, tannins, saponins, flavonoids, and hydrogen-bonded carboxylic acid [53,54] acting as reducing and stabilizing agents. Salvia species, including *S. greggii*, have been reported to contain a diverse array of bioactive phytochemicals such as catechin, gallic acid, kaempferol, apigenin, quercetin, naringin, hypericin, luteolin, pseudohypericin, hyperforin, glycosides, rosmarinic acid, carnosol, alpha-pinene, camphene, limonene, γ-terpinene, 1,8-cineol, camphor, and isobornyl acetate [21,28,30,33,34]. In particular, the leaf extract of *S. greggii* has demonstrated elevated levels of glycosidic flavones [31]. These phytochemicals are likely to play a crucial role in the reduction and stabilization of SB-AgNPs and SG-AgNPs. In addition, the presence of N–H stretches in the FTIR spectrum indicates the presence of amino acids, peptides, or nitrogen-containing aromatic compounds, as supported by Geremew et al. [17]. Furthermore, the observed C–H stretch, indicative of aliphatic and aromatic compounds, is suggestive of terpenoids serving as surface-protective functional groups [55]. Consistent with these findings, *S. greggii* leaf extract has been previously characterized for its rich terpenic compounds [56]. Overall, the identified functional groups, bonds, and linkages, as revealed through the FTIR analysis, play a critical and multifaceted role in maintaining the stability of synthesized SB-AgNPs and SG-AgNPs.

**Figure 3:**
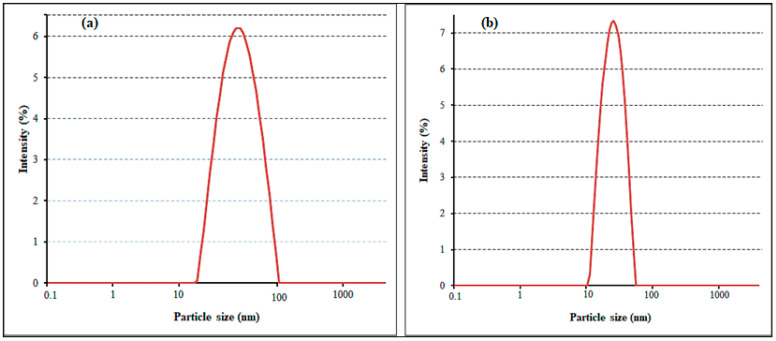
Particle size distribution of SB-AgNPs (**a**) and SG-AgNPs (**b**) from DLS analysis.

Particle size and surface charge were measured using the DLS method. The particle size distribution curve revealed that synthesized AgNPs were monodispersed in aqueous solution, with average diameters of 45.76 and 54.43 nm for SB-AgNPs and SG-AgNPs, respectively (Figure 3a,b). It is noteworthy that nanoparticles with sizes below 100 nm and a PDI less than 0.3 are recognized as being suitable for cellular uptake [57]. This characteristic is of particular significance for applications in the food safety and biomedical fields, owing to the nanoparticles’ capacity to efficiently penetrate cell membranes and disrupt microbial cells. The variation in particle size between SB-AgNPs and SG-AgNPs, despite employing the same synthesis method, can likely be attributed to differences in the phytochemical composition of SBLE and SGLE, as shown in FTIR.

**Figure 4:**
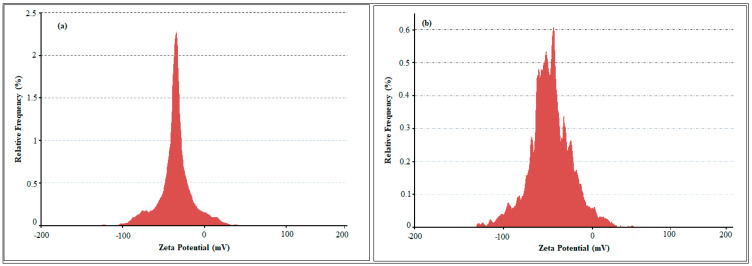
Zeta potential of SB-AgNPs (**a**) and SG-AgNPs (**b**) from DLS analysis.

The zeta potential is an indicator of surface charge potential which is an important parameter for understanding the stability of nanoparticles in aqueous suspensions. The measured zeta potential values of SB-AgNPs and SG-AgNPs were −23.2 mV and −25.8 mV, respectively (Figure 4a,b). In agreement with the present study, Gecer [21] has also reported a negative stable zeta potential (−20.3 mV) for AgNPs synthesized using leaf extract of the closely related species *S. aethiopis*. The zeta potential attains a value of zero at the iso-electric point, affirming the inherent instability [58]. In contrast, significantly negative or positive zeta potential values validate the robust stability of the synthesized nanoparticles in colloidal suspension. As highlighted in the FTIR analysis, the negative charge of SB-AgNPs and SG-AgNPs is likely due to the presence of phenolic compounds and specific amino acid residue side chains. The negative charge, the dissociation of some H^+^, and the total protonation of the hydroxyl groups of phytochemicals favor the repulsion between nanoparticles and ultimately reduce agglomeration.

**Figure 5:**
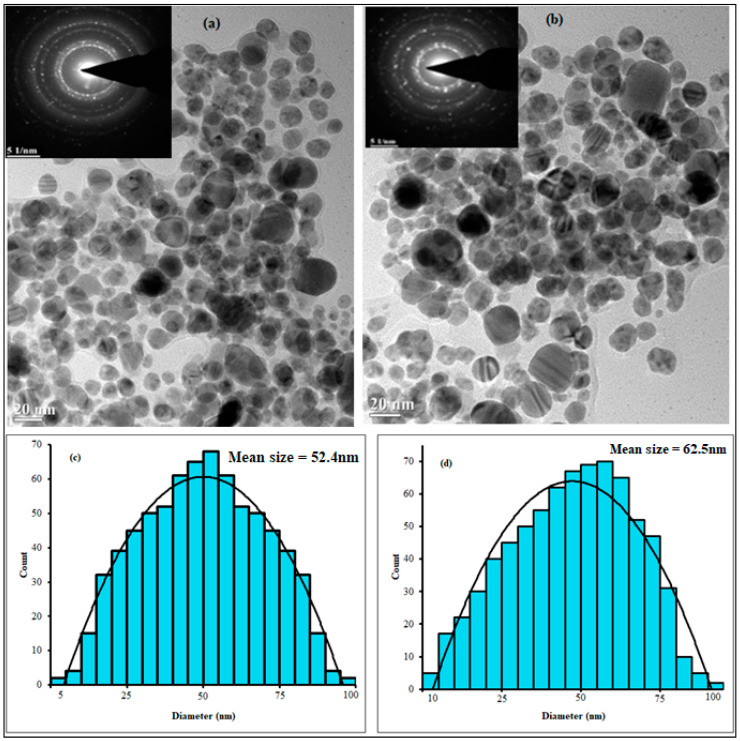
TEM images of SB-AgNPs (**a**) and SG-AgNPs (**b**) and their respective size distribution (**c**,**d**). Inset represents the selected area electron diffraction (SEAD).

The morphology, microstructure, and size distribution of the biosynthesized SB-AgNPs and SG-AgNPs were analyzed using high-resolution TEM. The TEM images clearly showed predominantly spherical SB-AgNPs and SG-AgNPs (Figure 5). The average particle size was 52.4 nm for SB-AgNPs and 62.5 nm for SG-AgNPs. Similarly, spherical-shaped AgNPs have been synthesized using leaf extracts from other closely related plant species such as *Salvia officinalis* [33,38,39,40] and *S. verticillate* [32]. However, the morphology of nanoparticles relies on the synthesis condition and the type of reducing and stabilizing agents used during fabrication [17,59].

**Figure 6:**
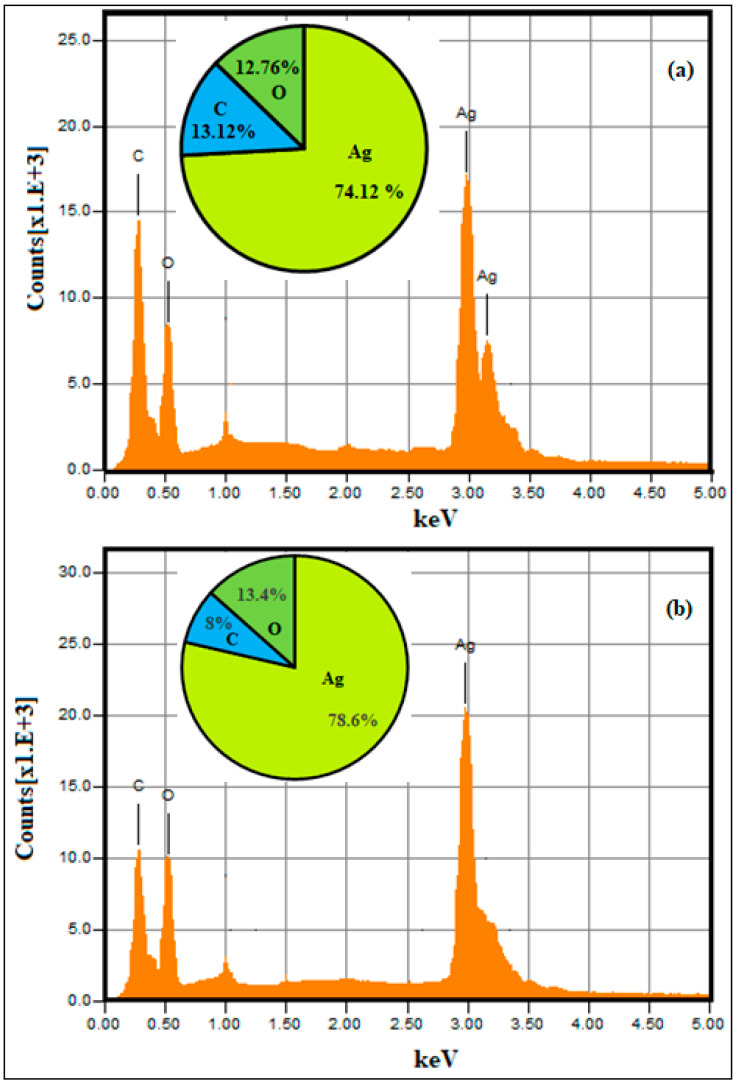
Elemental composition of SG-AgNPs (**a**) and SB-AgNps (**b**) from EDS analysis.

The qualitative and quantitative elemental composition of green-synthesized silver nanoparticles were examined using EDX spectroscopy. The EDX spectra of SB-AgNPs and SG-AgNPs confirmed the presence of 78.6% and 74.12% elemental silver along with the signal of 7.9% and 13.1% carbon and 13.4% and 12.6% oxygen, respectively (Figure 6a,b). For both biosynthesized nanoparticles, the analysis also revealed a strong peak at ~3 keV and weak signals of other organic compounds as carbon and oxygen at a range of 0–1.5 keV. Consistent with the findings from the FTIR results, the observed spectral signals corresponding to carbon and oxygen are ascribed to organic moieties such as carbonyl groups that act as capping and stabilizing agents of the nanoparticles [60,61].

**Figure 7:**
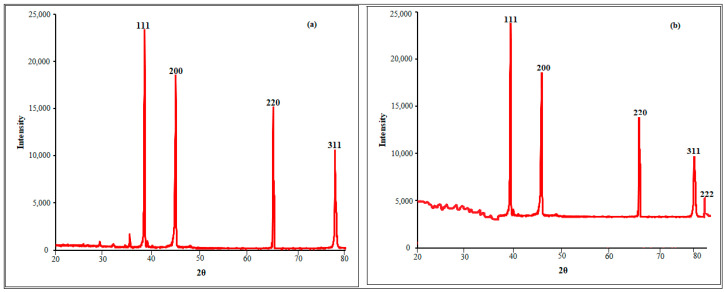
XRD patterns of biosynthesized AgNPs: SB-AgNPs (**a**) and SG-AgNPs (**b**).

The crystalline natures of the synthesized SB-AgNPs and SG-AgNPs were analyzed with XRD and provided in Figure 7a,b. The XRD pattern for SB-AgNPs revealed four diffraction peaks at 38.45°, 46.34°, 64.73°, and 78.03°, which correspond to the planes (111), (200), (220), and (311), respectively. On the one hand, XRD analysis revealed Bragg reflection values of SG-AgNPs—38.25°, 46.46°, 64.1°, 77.03°, and 81.64° 2θ angle—which corresponded to (111), (020), (022), (131), and (222), respectively. The Bragg peaks observed in both SB-AgNPs and SG-AgNPs spectra demonstrate the presence of a face-centered cubic (FCC) lattice structure in the AgNPs. This finding aligns with previously published works with the Joint Committee on Powder Diffraction Standards (JCPDS) file # 04-0783 [17,62,63,64]. The mean crystallite dimensions were determined to be 51.8 nm for SB-AgNPs and 60.9 nm for SG-AgNPs. This observed variation in crystallite sizes between SB-AgNPs and SG-AgNPs is further evidenced by a distinctive subtle peak at the (222) plane of SG-AgNPs, which is attributable to disparities in phytochemical compounds within the aqueous extract, as elucidated by Nayab and Akhtar [65]. 

### 2.2. Antibacterial Effects of Biosynthesized AgNPs on Foodborne Pathogens

**Figure 8:**
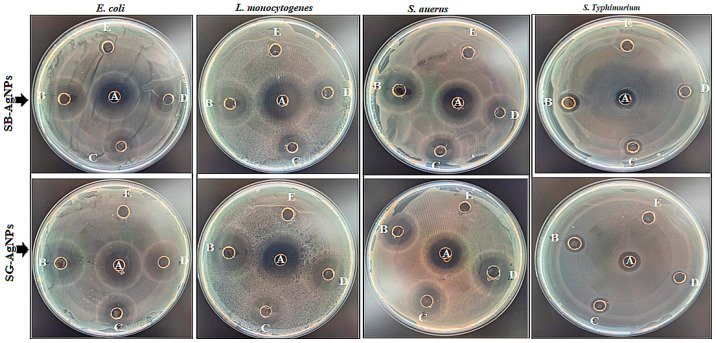
Zone of inhibition (mm) observed when *E. coli*, *L. monocytogenes*, *S. aureus*, and *S. typhimurium* were treated with SBLE, SGLE, SB-AgNPs, and SG-AgNPs at concentrations of 100 (B), 75 (C), 50 (D) μg mL^−1^, and the negative control streptomycin (A). The leaf extracts (SBLE and SGLE) are represented in (E).

The synthesized SB-AgNPs and SG-AgNPs were evaluated for their antibacterial activities against both Gram-positive and Gram-negative bacterial strains using agar well diffusion and MIC assays. The results showed that the synthesized SB-AgNPs and SG-AgNPs demonstrated antibacterial activity, displaying inhibition zones ranging from 9 to 19.2 mm and 7 to 20.5 mm, respectively, against the model foodborne pathogens used (Figure 8; Table 1). The most substantial antibacterial activity was observed against *L. monocytogenes*, displaying high sensitivity to both SB-AgNPs and SG-AgNPs with ZI values of 19.2 mm and 20.5 mm, respectively, at 100 μg mL^−1^. Following this, *S. aureus* exhibited ZI values of 16.3 mm and 17.6 mm, respectively, for SB-AgNPs and SG-AgNPs at the highest concentration. Despite the difference in bacterial response against each AgNPs, overall, there was significant comparable antimicrobial activity at higher concentrations with the control (*p* ≤ 0.05).

In addition, the MICs of synthesized AgNPs align with the observed antibacterial activity in the agar well diffusion test (Table 2). While the lowest MICs of 25.7 ± 3.2 μg mL^−1^ (SB-AgNPs) and 23.5 ± 2.5 μg mL^−1^ (SG-AgNPs) were recorded for *L. monocytogenes,* the highest MICs of 45.2 ± 5.3 μg mL^−1^ (SB-AgNPs) and 53 ± 3.6 μg mL^−1^ (SG-AgNPs) were observed for *S. typhimurium*. The same pattern was observed for the MBC test. Both the MIC and MBC were significantly different (*p* ≤ 0.05) across bacteria species under the SB-AgNPs and SG-AgNPs.

The antimicrobial sensitivity test revealed that the Gram-positive bacteria were found to be more sensitive than the Gram-negative bacteria to the SB-AgNPs and SG-AgNPs. Significant antibacterial activities have been reported for AgNPs synthesized using the closely related species *S. officinalis* [21]. The minimum magnitude of antibacterial activity against Gram-negative bacteria (*E. coli* and *S. typhimurium*) in the present study contrasts with several reports [17,43]. Nevertheless, the effectiveness of the antibacterial activity of AgNPs is intricately tied to their size and zeta potential, crucial factors determining their ability to penetrate the cell membrane and effectively kill the bacteria [44,66]. Specifically, smaller-sized SB-AgNPs and SG-AgNPs boast a larger surface area in comparison to their larger counterparts, resulting in heightened antibacterial efficacy [44,67]. Furthermore, the crucial role in the absorption of AgNPs that mainly cause microbial cell death is attributed to their spherical nature [68,69].

Silver nanoparticles have garnered substantial interest due to their remarkable antimicrobial properties, demonstrating efficacy against a diverse spectrum of microorganisms by employing various mechanisms. The uptake of silver ions dissociated from AgNPs by bacteria triggers a sequence of events that disrupt crucial cellular functions. They interact with microbial DNA, inducing structural modifications that hinder DNA replication and transcription [70]. Additionally, AgNPs have been observed to interfere with the enzymatic operations of vital microbial enzymes, such as glucose dehydrogenase. Moreover, studies have revealed that AgNPs can cause the denaturation or functional impairment of proteins [44,71]. This impairment significantly disrupts essential cellular processes and metabolic pathways, thereby negatively impacting overall microbial function [72]. Nanoparticles have been reported to inhibit the respiratory chain in microorganisms, disrupting energy production by interfering with the microbial electron transport chain, ultimately culminating in cell death [55,60,73,74]. Conversely, AgNPs have been found to induce the generation of free radicals and reactive oxygen species (ROS) that potentially cause oxidative stress and inflict damage on microbial DNA, proteins, and lipids [17,75,76]. Moreover, AgNPs can interact with the cell membranes of microorganisms, disrupting their structural integrity, and this leads to increased permeability, leakage of cellular contents, and ultimately cell death in different foodborne pathogens [17,43,76,77,78]. The growth inhibition caused by the AgNPs is primarily due to the formation of pores in the bacterial cell wall accompanied by changes in cell membrane permeability due to the deposition of AgNPs at critical sites [60,79,80]. Moreover, AgNPs interact with the microorganism cell membranes, disrupting their structure, increasing permeability, causing content leakage, and leading to cell death in various foodborne pathogens [17,43,44,76]. Mechanistically, the inhibitory effect of these nanoparticles might also be attributed to the disruption of thiol groups in the electron transport chain enzymes, followed by the binding of AgNPs to the cell wall and membrane of bacterial cells [43,44]. Overall, the broad-spectrum antibacterial activity of the synthesized AgNPs is due to combinatorial action [43,80,81].

### 2.3. Antioxidant Activity of SB-AgNPs and SG-AgNPs

**Figure 9:**
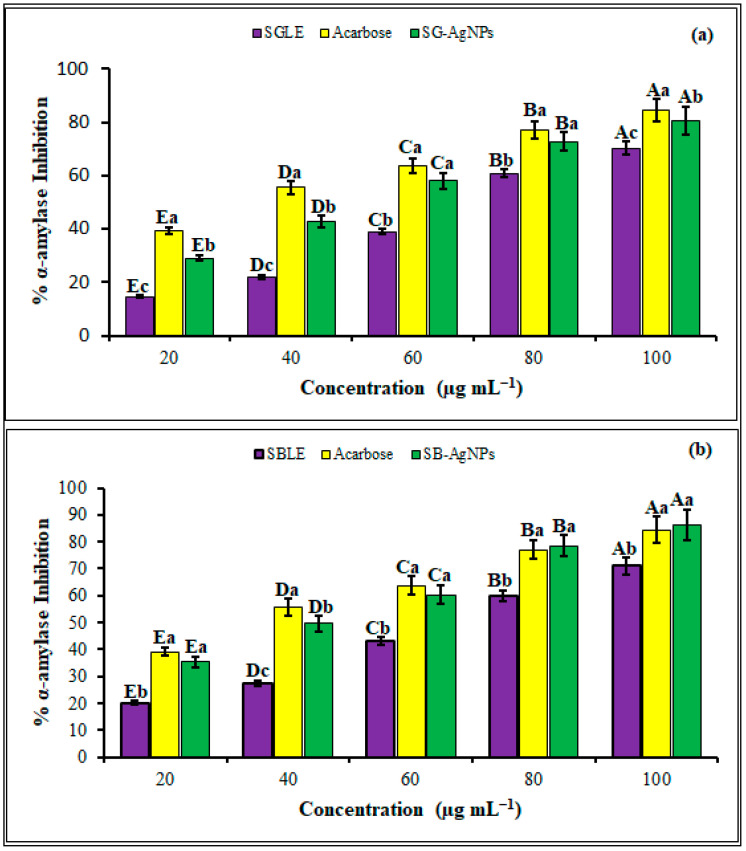
The antioxidant activities of *S. greggii* leaf extract (SGE) and SG-AgNPs (**a**) and *S. blepharophylla* leaf extract (SBE) and SB-AgNPs (**b**) at different concentrations using DPPH radical scavenging assay. Different lower-case letters denote significant differences (*p* ≤ 0.05) among Acarbose, SGLE, or SBLE and SG-AgNPs or SB-AgNPs at a given concentration, while uppercase letters denote significant differences across different concentrations of a particular antioxidant. AA indicates ascorbic acid. Values are reported as the mean ± standard deviation.

Oxidative stress, stemming from cellular oxidation and the subsequent production of free radicals and ROS, is widely recognized as a significant factor in various health conditions, including rheumatoid arthritis, diabetes, cancer, aging, cardiovascular diseases, and neurodegenerative diseases [82,83]. The excessive presence of these radicals can detrimentally impact the defensive antioxidant enzymes, potentially resulting in cellular injury or apoptosis [84]. In the current study, the antioxidant activities of the green-synthesized SB-AgNPs and SG-AgNPs were evaluated using DPPH. The DPPH scavenging activity of SB-AgNPs, SG-AgNPs, SBLE, SGLE, and the standard (ascorbic acid) is shown in Figure 9. The antioxidant properties increased significantly with increasing concentrations of SB-AgNPs and SG-AgNPs in a dose-dependent pattern (*p* ≤ 0.05). The DPPH scavenging activity of SB-AgNPs ranged between 45.3% and 90.2%, whereas SG-AgNPs activity ranged between 45.2% and 89.5%. Nevertheless, the scavenging potential of the SB-AgNPs and SG-AgNPs was comparatively higher than the standard at a μg mL^−1^ concentration by 3.2% and 2.4%, respectively. SB-AgNPs and SG-AgNPs exhibited the highest antioxidant activity—90.2% and 89.5%, respectively—at 100 μg mL^−1^.

The antioxidant properties of AgNPs may be attributed to the varied functional groups (Figure 2) originating from *S. blepharophylla* and *S. greggii* leaf extracts that are responsible for the bioreduction of Ag^±^ ions to AgNPs. The antioxidant mechanisms of AgNPs are diverse and intricate, offering various strategies to combat oxidative stress and minimize cellular damage [17]. The developed nanoparticles demonstrate multifaceted antioxidant properties that might directly scavenge ROS and free radicals, thereby mitigating oxidative stress and cellular harm [85,86]. They may also enhance the activity of endogenous antioxidant enzymes, such as superoxide dismutase, catalase, and glutathione peroxidase, improving ROS neutralization [87]. Additionally, AgNPs can chelate transition metal ions like iron and copper, known for their involvement in generating highly reactive hydroxyl radicals in Fenton reactions, thus reducing oxidative stress [88]. Moreover, these nanoparticles might indirectly improve cellular antioxidant defense by inhibiting lipid peroxidation, elevating intracellular glutathione levels, and maintaining the redox balance within cells [87,89,90,91,92]. However, it is crucial to note that the antioxidant properties of AgNPs vary depending on factors such as their size, shape, surface chemistry, and the specific biological system under examination [17].

### 2.4. Antidiabetic Activities of SB-AgNPs and SG-AgNPs

**Figure 10:**
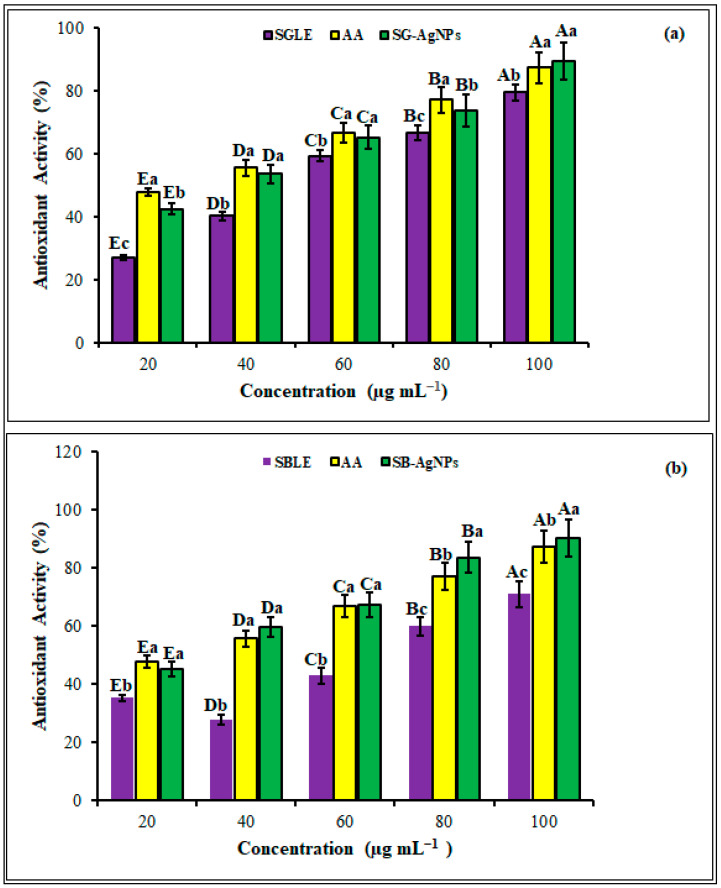
The antidiabetic activities of *S. greggii* leaf extract (SGLE) and SG-AgNPs (**a**) and *S. blepharophylla* leaf extract (SBLE) and SB-AgNPs (**b**) at different concentrations using α-amylase inhibition assay. Different lowercase letters denote significant differences (*p* ≤ 0.05) among Acarbose, SGLE, or SBLE and SG-AgNPs or SB-AgNPs at a particular concentration, while uppercase letters denote significant differences across different concentrations of a particular antidiabetic agent. Acarbose was used as a standard. Values are reported as the mean ± standard deviation.

Diabetes mellitus encompasses a set of metabolic illnesses distinguished by continuously elevated blood sugar levels (hyperglycemia). One effective approach to managing postprandial hyperglycemia-linked Type II diabetes involves the inhibition of carbohydrate-digesting enzymes like α-amylase. The exploration of nanoparticles that inhibit carbohydrate hydrolyzing enzymes, as an alternative antidiabetic material, is a continuing effort [92,93,94,95,96]. In the present study, both SB-AgNPs and SG-AgNPs were evaluated for their inhibition potential against carbohydrate-metabolizing enzyme α-amylase at different concentrations. Both SB-AgNPs and SG-AgNPs demonstrated significant concentration-dependent antidiabetic activity (*p* ≤ 0.05). The α-amylase inhibition potential of SB-AgNPs varied between 35.4% and 86.5%, while, for SG-AgNPs, it ranged between 29% and 80.5% in a dose-dependent manner (20–100 μg mL^−1^) (Figure 10). The standard (acarbose), SB-AgNPs, and SG-AgNPs showed a maximum α-amylase inhibition of 84.4%, 86.5%, and 80.5%, respectively, at 100 μg mL^−1^. Concurring with our results, several studies have reported the anti-diabetic activity of AgNPs using an α-amylase inhibition assay [97,98,99,100,101].

The antidiabetic potential of SB-AgNPs and SG-AgNPs appears to be linked with their demonstrated antioxidant properties. Research has consistently underscored the pivotal role of antioxidants in diabetes management by reducing oxidative stress and improving critical aspects of the condition such as insulin sensitivity, glycemic control, and the prevention of complications [85,102,103,104]. The mechanism by which AgNPs operate involves the scavenging of ROS and free radicals along with the upregulation of endogenous antioxidant enzymes. This concerted action aids in the reduction of oxidative stress, a significant contributor to the onset and advancement of diabetes [85,87]. Moreover, apart from the active site and enzyme–substrate complex, AgNPs inhibit enzyme catalytic activity by binding to the non-active site of the α-amylase [97]. Overall, our comprehensive findings highlight the potential of SB-AgNPs and SB-AgNPs as viable candidates for the development of novel antidiabetic agents or for improving existing antidiabetic medications, particularly upon conducting a thorough investigation into their molecular-level mechanisms of action.

## 3. Materials and Methods

### 3.1. Plant Samples and Chemicals

Leaves from two medicinal plants, *Salvia blepharophylla* and *Salvia greggii*, were collected from John Fairey Garden, Hempstead, Texas. Ethanol, silver nitrate, 1,1-diphenyl-2-picrylhydrazyl (DPPH), acarbose, starch, ascorbic acid, and methanol were obtained from Sigma Aldrich (99.9%, St-Louis, MO, USA). Tryptic soy broth (TSB) and agar (TSA) were supplied by Becton–Dickinson (Franklin Lakes, NJ, USA). *Escherichia coli* O157:H7 (ATCC 43888), *Staphylococcus aureus* (ATCC 6538), *Listeria monocytogenes* (4b) (ATCC 19115), and *Salmonella typhimurium* (ATCC 14028) were purchased from Fisher Scientific (Hampton, VA, USA).

### 3.2. Preparation of Plant Extracts

The young and green leaves of *S. blepharophylla* and *S. greggii* collected were cleaned by rinsing in distilled water several times. Then, leaves were freeze-dried using a BenchTop Pro with Omnitronics™ freeze dryer (BTP-8ZL00W, SP Scientific, Warminster, PA, USA) and were grinded using an electrical blender. Subsequently, the leaf powder (5 g each) was added to flasks containing 500 mL Milli-Q water (18.2 MΩ cm) and was shaken with an orbital shaker (IKA Basic Variable-Speed Digital Orbital Shaker, model, 115 V) at 200 rpm at 35 °C for 72 h followed by sonication using an ultrasonic bath (Bransonic^®^ MH-series, CPX-952-217R, Danbury, CT, USA) for 90 min and maceration for 2 h at room temperature. The extract solution was filtered using a Stericup^®^ Quick Release Vacuum-driven disposable filter integrated with Millipore Express^®^ Plus 0.22 µm PES System (Sigma Aldrich, St-Louis, MO, USA). The filtrates were kept at 4 °C pending the synthesis of AgNPs.

### 3.3. Green Synthesis of Silver Nanoparticles

The green synthesis of AgNPs was performed following the procedure reported in our previous study [17]. Briefly, 5 mL each of *S. blepharophylla* and *S. greggii* leaf extracts (SBLE and SGLE, respectively) were mixed with 45 mL of 0.1 mM of AgNO_3_ aqueous solution under continuous stirring at room temperature for the reduction of Ag ions to form SB-AgNPs and SG-AgNPs, respectively. The solutions were stirred overnight in the dark until the complete synthesis of nanoparticles had occurred, as indicated by a color change from light yellow to dark brown. Afterwards, the colloidal mixtures were shaken thoroughly and centrifuged at 10,000 rpm for 20 min. The nanoparticle pellets were collected by decanting the supernatant, were dried at room temperature, and were stored in dark vials pending further characterization.

### 3.4. Characterization of SB-AgNPs and SG-AgNPs

SB-AgNPs and SG-AgNPs were successfully synthesized using SBLE and SGLE as starting material. The formation of SB-AgNPs and SG-AgNPs in aqueous suspension was determined using a UV-Vis spectrophotometer (SpectraMax^®^ PLUS, Molecular Devices, San Jose, CA, USA) during the reaction and after the completion of synthesis, respectively, to observe peaks between 200 and 750 nm. The stability of the nanoparticles was determined using absorption spectra obtained for 30 min, 2 h, 3 h, 24 h, 30 days (724 h), and 36 days (869 h). The dynamic light scattering method was used to estimate the particle size distribution, polydispersity index and zeta potential using a Litesizer^TM^ 500 (Anton Paar, Graz, Austria) coupled with a 10 mW He-Ne laser (633 nm) running at an angle of 90° at 20 °C. The zeta potential values influence the stability of nanoparticles in the colloidal system and facilitate the understanding of interparticle forces of interaction [43]. The average of the zeta-potential values was computed from three independent measurements. The Fourier transform infrared (FTIR, JASCO/FTIR-6300, Tokyo, Japan) spectra of biogenic nanoparticles (SB-AgNPs and SG-AgNPs), SBLE and SGLE, were obtained in order to identify functional groups. The samples were analyzed at 4 cm^−1^ resolution and a wavelength between 500 and 4000 cm^−1^.

A high-resolution transmission electron microscope (TEM, JEOL-2100, Peabody, MA, USA) was used to identify the depth morphological features, size distribution, and selected area electron diffraction (SAED) of SB-AgNPs and SG-AgNPs at an accelerating voltage of 200 kV. Additionally, the elemental composition of SB-AgNPs and SG-AgNPs was analyzed using energy-dispersive X-ray spectroscopy (EDS, JOEL JSM-6010 LA, Peabody, MA, USA). The phase purity, crystallinity, and size of biologically synthesized AgNPs were analyzed using an X-ray diffractometer (XRD-7000, Shimadzu, Kyoto, Japan) operated at 40 kV with a current of 30 mA and Cu-Ka radiation at a 1.514 Å wavelength. The spectrum was recorded in the 2θ range of 20° to 80° with a step size of 0.3 s.

### 3.5. Antibacterial Activity

#### 3.5.1. Sensitivity Test

Experiments on antimicrobial activity were performed following the Institute for Clinical and Laboratory Standards [105]. The antibacterial activities of SB-AgNPs and SG-AgNPs were investigated on Gram-positive (*S. aureus* ATCC 12228 and *L. monocytogenes* ATCC 19111) and Gram-negative (*E. coli* 0157:H7 and *S. typhimurium* ATCC 14028) bacterial strains using the well-diffusion method. Initially, for inoculum preparation, bacteria were cultured in TSA media (Becton–Dickinson, Franklin Lakes, NJ, USA) by incubating for 24 h, and then isolated colonies were inoculated in TSB (Becton–Dickinson, Franklin Lakes, NJ, USA) to make suspensions. The turbidity of the suspension was adjusted to achieve a CFU of 1.0–2.0 × 10^6^ CFU mL^−1^ using a UV–Vis Spectrophotometer (SpectraMax^®^ PLUS, Molecular Devices, San Jose, CA, USA) with a 600 nm bacterial culture suspension (0.1 mL of each) inoculated on TSA plates and uniformly spread with a sterile spreader. The growth media with bacteria were punctured using a sterile glass borer to make five wells (6 mm each) in each plate equidistant from one another. Then, 125 μL of 50, 75, and 100 μg mL^−1^ of the synthesized SB-AgNPs and SG-AgNPs, 100 μg mL^−1^ each of SBLE and SGLE, and 100 μg mL^−1^ each of streptomycin (positive control) were pipetted to individual wells. The prepared plates were then incubated for 24 h at 37 °C. After incubation, the diameters of the inhibition zone (DZI) were measured using ProtoCOL3 (Synbiosis, Cambridge, UK). This experiment was run in triplicates.

#### 3.5.2. Minimum Inhibitory and Minimum Bactericidal Concentrations of SB-AgNPs and SG-AgNPs

The minimum inhibitory concentrations (MICs) of the SB-AgNPs and SG-AgNPs were determined using the broth micro-dilution method. The bacteria were cultured to prepare 0.5 McFarland turbidity standard (A600 = 0.1), corresponding to 1 × 10^6^ CFU/mL. Different concentrations of SB-AgNPs and SG-AgNPs were prepared using sterilized water and were added to the microtiter wells to obtain the final concentrations of 5, 10, 20, 25, 30, and 35 μg mL^−1^. TSB media (100 µL), 1 × 10^6^ bacterial culture (20 µL), and the different concentrations of SB-AgNPs and SG-AgNPs (80 µL) were added in each well of the 96-well microplate [17] in order to acquire a final volume of 200 µL in each well. Further, the microplates were incubated at 30 °C for 24 h. For each dilution series, an equal amount (80 µL) of SB-AgNPs and SG-AgNPs each and TSB (no bacterial inoculums) were used as negative controls while untreated cell suspensions (only medium and bacteria inoculums) were administered as positive controls. The MIC values were identified as the minimum concentration at which no visible bacterial growth was recorded. On the other hand, the minimum bactericidal concentration (MBC) of SB-AgNPs and SG-AgNPs was evaluated by subculturing bacteria with a concentration equal to or higher than MIC on TSA plates and incubating at 30 °C for 24 h. The lowest concentration that did not exhibit bacterial growth was defined as the MBC value. All the experiments were carried out in triplicates.

### 3.6. Evaluation of Antioxidant Assay

The antioxidant activities of synthesized SB-AgNPs and SG-AgNPs, SBLE, and SGLE were determined by 2, 2-diphenyl-1-picrylhydrazyl (DPPH) radical scavenging assay with a slight modification [17]. SB-AgNPs, SG-AgNPs, SBLE, SGLE, and ascorbic acid (control) were prepared at varying concentrations of 20, 40, 60, 80, and 100 μg mL^−1^, and 30 μL were added to each well of a 96-well microplate. Subsequently, the solution was mixed with 170 μL of 0.1 mM DPPH and shaken thoroughly. The solution was incubated in the dark for 20 min. The decrease in the concentrations of DPPH was monitored by measuring absorbance at 517 nm using UV-Vis spectrophotometry. DPPH in methanol without SBLE, SGLE, SB-AgNPs and SG-AgNPs was used as a control. Later, the antioxidant capacities of the nanoparticles and the leaf extracts were expressed in % as follows:

% Radical scavenging (DPPH) = [(Ao − A1)/Ao] × 100 (1)

where Ao represents the OD of either ascorbic acid or the leaf extracts, and A1 denotes the OD of the nanoparticles after reacting with DPPH.

### 3.7. Antidiabetic Activities of SB-AgNPs and SG-AgNPs

The anti-hyperglycemic activities of SB-AgNPs and SG-AgNPs were examined by evaluating the inhibition of α-amylase following Sathiyaseelan et al. [106] with minor modification. Twenty (20) μL of different concentrations of SB-AgNPs and SG-AgNPs (20, 40, 60, 80, and 100 μg mL^−1^) were added to 15 μL of α-amylase (6 unit mL^−1^), and the volume was adjusted to 100 μL with 1 mM phosphate buffer (pH 6.5). Then, the mixture was pre-incubated at 37 °C for 15 min, and 25 μL of starch solution (1% *w*/*v*) was added. The reaction mixture was incubated at 75 °C in a water bath for 10 min, and then, the reaction was stopped by using 65 μL of 0.1 M of dinitrosalicylic acid reagent (DNSA). The control was prepared without the addition of nanoparticles and acarbose was employed for positive control. After cooling the reaction mix, the α-amylase inhibitory activities of the nanoparticles were measured by recording the absorbance values at 450 nm using UV-Vis spectrophotometer. For each biosynthesized nanoparticle, percentage inhibition of α-amylase was computed as follows:

Inhibitory activity (%) = [(Ac − At)/Ac] × 100 (2)

where At is the absorbance of the test sample, and Ac is the absorbance of the control.

### 3.8. Statistical Analysis

One-way analysis of variance (ANOVA) followed by Tukey’s multiple comparisons post hoc was used for statistical analysis using the SPSS 17.0 software package (SPSS, Inc., Chicago, IL, USA). Three replications for each of the experiments and assays were conducted. The values reported in the result sections were the mean and standard error (±SE) of triplicates. Statistical significance was acceptable to a level of *p* < 0.05.

## 4. Conclusions

In conclusion, the study elucidated the green synthesis of silver nanoparticles (SB-AgNPs and SG-AgNPs) using *Salvia blepharophylla* and *Salvia greggii* leaf extracts. The UV-Vis spectrum analysis revealed robust and broad-spectrum absorbance with a pronounced SPR peak at approximately 420 nm for both SB-AgNPs and SG-AgNPs. Fourier transform infrared (FTIR) analysis identified various functional groups in the leaf extracts and nanoparticles, suggesting the involvement of phytochemicals as reducing and stabilizing agents. Particle size and surface charge analysis revealed monodispersed nanoparticles with average diameters suitable for cellular uptake. High-resolution TEM images displayed predominantly spherical nanoparticles with average sizes of 52.4 nm for SB-AgNPs and 62.5 nm for SG-AgNPs. The antibacterial activities of SB-AgNPs and SG-AgNPs were evaluated against Gram-positive and Gram-negative bacteria, showing inhibition zones and minimum inhibitory concentrations. The synthesized nanoparticles exhibited significant antibacterial efficacy, with Gram-positive bacteria being more sensitive than Gram-negative bacteria. Additionally, the DPPH scavenging assays on both nanoparticles exhibited dose-dependent antioxidant properties, surpassing the standard (ascorbic acid) at the highest concentration. Furthermore, the SB-AgNPs and SG-AgNPs exhibited improved anti-diabetic action against α-amylase. Overall, the study suggests that the synthesized SB-AgNPs and SG-AgNPs possess multi-faceted biological activities, making them promising candidates for applications in antimicrobial, antioxidant, and antidiabetic fields. Further research is required to unravel the detailed molecular mechanisms underlying these activities and to explore their potential applications in various biomedical and pharmaceutical contexts.

## Figures and Tables

**Table 1:** Antimicrobial sensitivity in terms of zone of inhibition diameter (mm) observed when foodborne pathogens were treated with SBLE, SGLE, SB-AgNPs, and SG-AgNPs and the control streptomycin. Different letters in the same column indicated that values were significantly different at *p* ≤ 0.05. Values are indicated as mean ± standard error.

Bacteria	Streptomycin	SBLE	SB-AgNPs			SGLE	SG-AgNPs		
			100	75	50		100	75	50
*E. coli*	16 ± 1.8 ^ab^	11.2 ± 1.1 ^b^	14 ± 1.3 ^bc^	13 ± 1.3 ^bc^	13 ± 1.2 ^bc^	11.3 ± 1.1 ^b^	16 ± 1.4 ^ab^	15 ± 1.7 ^ab^	12 ± 1.2 ^b^
*S. typhimurium*	11 ± 1.5 ^b^	6.5 ± 0.7 ^c^	11 ± 1.1 ^b^	10 ± 1.0 ^b^	9 ± 0.9 ^bc^	6.3 ± 0.7 ^c^	12 ± 0.9 ^b^	9 ± 0.8 ^bc^	7 ± 0.6 ^c^
*S. aureus*	18 ± 1.7 ^a^	6.2 ± 0.6 ^c^	16.3 ± 1.5 ^ab^	15 ± 1.2 ^b^	14 ± 1.2 ^ab^	6.4 ± 0.5 ^c^	17.6 ± 1.7 ^a^	16 ± 1.3 ^ab^	15 ± 1.5 ^ab^
*L. monocytogenes*	20 ± 1.8 ^a^	6.2 ± 0.7 ^c^	19.2 ± 1.5 ^a^	16 ± 1.4 ^ab^	15 ± 1.4 ^ab^	6.5 ± 0.6 ^c^	20.5 ± 1.8 ^a^	17 ± 1.5 ^ab^	15 ± 0.9 ^ab^

**Table 2:** Antibacterial activity of green-synthesized SB-AgNPs and SG-AgNPs measured in terms of minimum inhibitory/bactericidal concentration (MIC/MBC) for different foodborne pathogens. Different letters in the same column indicated that values were significantly different at *p* ≤ 0.05. Values are indicated as the mean ± standard error.

Bacteria	SB-AgNPs (μg mL^−1^)		SG-AgNPs (μg mL^−1^)	
	MIC	MBC	MIC	MBC
*E. coli*	39 ± 4.1 ^ab^	83 ± 6.1 ^b^	40 ± 4.1 ^b^	78.5 ± 5.4 ^b^
*S. typhimurium*	45.2 ± 5.3 ^a^	98 ± 5.1 ^a^	53 ± 3.6 ^a^	88.5 ± 7.2 ^a^
*S. aureus*	32.4 ± 2.5 ^b^	54 ± 3.6 ^c^	30 ± 3.2 ^c^	50.2 ± 4.2 ^c^
*L. monocytogenes*	25.7 ± 3.2 ^c^	45 ± 3.2 ^d^	23.5 ± 2.5 ^d^	42.7 ± 3.1 ^d^

## Data Availability

Any data supporting the findings of this article will be made available by the authors without undue reservation.
